# The Effects of Dietary Supplementation with Collagen and Vitamin C and Their Combination with Hyaluronic Acid on Skin Density, Texture and Other Parameters: A Randomised, Double-Blind, Placebo-Controlled Trial

**DOI:** 10.3390/nu16121908

**Published:** 2024-06-17

**Authors:** Katja Žmitek, Janko Žmitek, Hristo Hristov, Mirjam Rogl Butina, Petra Keršmanc, Tina Pogačnik

**Affiliations:** 1 VIST—Faculty of Applied Sciences, Institute of Cosmetics, Gerbičeva ulica 53, 1000 Ljubljana, Slovenia; janko.zmitek@vist.si (J.Ž.); mirjam.rb@dermatologija.eu (M.R.B.); petra.kersmanc@vist.si (P.K.); tina.pogacnik@vist.si (T.P.); 2 Nutrition Institute, Koprska ulica 98, 1000 Ljubljana, Slovenia; hristo.hristov@nutris.org; 3 Dermatologija Rogl Fabjan (Rogl Fabjan Dermatology), Derčeva ulica 35, 1000 Ljubljana, Slovenia

**Keywords:** collagen, hyaluronic acid, ageing skin, density, texture, wrinkles

## Abstract

Collagen dietary supplements are becoming increasingly popular as a means to reduce signs of skin ageing. The objective of this three-way, randomised, placebo-controlled, double-blind study was to examine and contrast the effects of dietary supplementation with a daily dose of 5 g hydrolysed collagen with 80 mg of vitamin C (CP product) and their combination with 30 mg of hyaluronic acid (CPHA product) over 16 weeks. Validated methods were utilised for the objective evaluation of skin parameters. In total, 87 subjects (women, 40–65 years) completed the entire trial, distributed across the groups as follows: placebo group (*n* = 29), CPHA group (*n* = 28), and CP group (*n* = 30). The results showed beneficial effects of both test products, with notable enhancements in dermis density, skin texture, and a reduction in the severity of wrinkles. In contrast, the administration of either of the products did not yield any significant impacts on skin elasticity or hydration. Observation of the investigated skin parameters did not show superior effects of the addition of hyaluronic acid (HA) to collagen. Therefore, the ability of supplementation with HA to improve the effects on investigated skin parameters beyond the supplementation of collagen alone cannot be confirmed.

## 1. Introduction

The skin is our largest organ, serving as a crucial barrier protecting the body against the environment; alterations in the skin’s appearance and properties that occur with age often manifest the first visible signs of ageing [1,2]. With global demographic changes and an ageing population [3], there is a shifting focus towards understanding the mechanisms of skin ageing, emphasising management strategies for achieving a more youthful appearance and overall well-being [4]. This shift is driven, in part, by the recognition that several factors notably affect ageing processes within the human body, particularly the skin. The intricate process of skin ageing results from an interplay of intrinsic ageing due to genetic factors and extrinsic ageing, which is influenced by environmental variables, including ultraviolet radiation exposure and nutrition [5,6].

Intrinsic and extrinsic ageing, despite their distinct morphological and pathological characteristics, exhibit several molecular commonalities. The generation of reactive oxygen species and the activation of matrix metalloproteinases (MMPs) are key factors in the process of skin ageing. Collagen, a pivotal protein in the dermis, plays a crucial role in maintaining skin physiology by providing strength and elasticity, while hyaluronic acid (HA), the predominant mucopolysaccharide in the skin, plays a crucial role in maintaining water balance and contributing to the skin’s structure [7]. However, with the progression of skin ageing, increased expression of MMPs leads to the deterioration of collagen and HA, alongside other extracellular matrix (ECM) components. The build-up of broken collagen fibres hinders the synthesis of new collagen and contributes to the continued breakdown of ECM through positive feedback control [1,8,9,10]. At the same time, the diminishing activity of fibroblasts hinders its synthesis, and the loss of HA, collagen and other ECM constituents results in reduced dermal volume, elasticity and hydration.

Recognising the link between nutrition and skin health, including all its possible aspects, from beauty and integrity to the ageing process, has led to a growing interest in dietary interventions and supplementation for a more youthful appearance [11,12]. Collagen supplementation has gained popularity due to its potential to stimulate the synthesis of ECM constituents, including collagen and HA, and inhibit their degradation by MMPs, making it a promising ingredient for combating skin ageing [13,14,15]. Multiple studies have examined the impact of collagen peptides on skin condition [7,16,17,18,19]. Most of these studies, which used a combination of collagen peptides, employed a blend of collagen peptides alongside other bioactive compounds. HA has also been studied as a possibly beneficial bioactive component for skin health. In trials, the consumption of HA showed promising results in improving various skin conditions, specifically hydration and signs of ageing; however, these were conducted mostly in Asian populations, and data concerning the impact of HA supplementation on the skin of Caucasians are scarce.

The main objective of the present intervention study was to investigate whether the effects of 16 weeks of supplementation with 5g of hydrolysed collagen with 80 mg of vitamin C (HC) on skin parameters could be further improved by supplementation with 30 mg of hyaluronic acid (HA). The primary focus of the study was on the impact of the intervention on dermal density, while a variety of other skin parameters were also followed. We also explored whether the effects of the tested interventions are comparable to supplementation with a higher dosage of collagen or the combination of collagen with methylsulphonylmethane (MSM), which was explored in our previous study [20].

## 2. Materials and Methods

### 2.1. Study Design

The research was performed at a single centre to assess the effects on the skin of 16 weeks of daily oral administration of one of the two experimental products or a placebo. The trial was randomised and double-blind, and a parallel three-way intervention design was used.

### 2.2. Study Population

In total, 90 healthy women aged 40–65 years were enrolled in the study. Their eligibility was assessed against the inclusion and exclusion criteria, which are in detail listed in the published study protocol [21]. All individuals included in the study provided written informed consent (ICF) prior to their involvement. They were enrolled on a consecutive basis using random assignment to one of three study groups, with 30 participants per group. Randomisation was accomplished through a simple randomisation procedure that utilised computer-generated random numbers using the ‘RAND’ function in Microsoft^®^ Excel^®^ for Microsoft 365 MSO (v16.0) using equal distribution between study groups.

### 2.3. Study Products and Intervention

The study was conducted at VIST–Faculty of Applied Sciences (Slovenia) throughout the winter-summer season (from February 2023 to July 2023).

The subjects consumed 15 mL of syrup per day with a meal over a period of 16 weeks. One study group (the CPHA group) was given the investigational product CPHA (daily dose 15 mL: collagen 5 g, hyaluronic acid: 30 mg, vitamin C: 80 mg); the second study group (the CP group) received the investigational product CP (daily dose 15 mL: collagen 5 g, vitamin C: 80 mg); and the third study group was given a placebo product that contained none of these active ingredients (15 mL: 0 g collagen, 0 g hyaluronic acid, 0 g vitamin C). The other ingredients in all three study products were water; acids: lactic, malic, and phosphoric; flavourings; sweeteners: xylitol, sucralose; preservatives: sodium benzoate, potassium sorbate. The placebo product also contained colour: caramel; stabilisers: xanthan gum and gellan gum.

The products were packaged in non-transparent bottles with a 500 mL capacity, ensuring that both the subjects and the researchers remained unaware of the specific product assigned to each subject. Each participant received a measuring cup that enabled convenient dosing of 15 mL of syrup.

Study products in syrup form were produced by TOSLA d.o.o. (Ajdovščina, Slovenia) according to food standards, maintaining strict control over heavy metals, microbiology, viscosity, pH, colour, taste, and odour. Hydrolysed bovine collagen with an average molecular weight of 2–4 kDa and hyaluronic acid with a molecular weight of 75–1500 kDa, produced through fermentation using *Streptococcus zooepidemicus*, were used in the products. At the end of the study, any remaining test products were required to be returned.

### 2.4. Assessments

The participants underwent regular checkups at three distinct stages: at baseline (T0), after eight weeks (T8), and after 16 weeks of supplementation (T16). To monitor adherence to the study protocol, the participants maintained a detailed log of their study product intake throughout the entire 16-week intervention period. This log was reviewed during the follow-up consultations at T8 and T16. Additionally, the participants were asked to document any instances of non-compliance.

Measurements of dermis density, thickness, skin hydration, and viscoelasticity were conducted at three time points, T0, T8, and T16, while skin roughness and wrinkles were assessed at two time points, T0 and T16. The measurements were conducted in a controlled atmosphere with a temperature of 20–25 °C and relative humidity of 40–60% while the subjects were in a supine position (or seated for roughness and wrinkle measurements). The assessments began after a 30-min period of acclimatisation under stable air conditions. The participants were provided with instructions to cleanse the measuring regions a minimum of 2 h before the measurement and to refrain from applying any cosmetic items to those areas within 2 h of the evaluation. To ensure the accuracy of the data, the measuring equipment underwent frequent calibration in accordance with the instructions provided by the manufacturer.

#### 2.4.1. Dermal Density and Thickness

The DermaLab Series SkinLab Combo 20 MHz ultrasound probe (Cortex Technology ApS, Aalborg, Denmark) was utilised to perform ultrasonography measurements of dermis density (0–100 intensity score) and thickness (µm). The measurements were performed in a designated region (approximately 4 cm^2^) on the right cheek using a constant gain curve for each individual. The measurements were taken twice, and the mean was computed.

#### 2.4.2. Skin Viscoelasticity

The DermaLab Series SkinLab Combo elasticity probe (Cortex Technology ApS, Aalborg, Denmark) was utilised to perform measurements of skin viscoelasticity (VE). The measurements were performed in a designated region (approx. 4 cm^2^) on the right cheek. The measurements were taken three times, and the mean was computed.

#### 2.4.3. Skin Hydration

The DermaLab Series SkinLab Combo hydration flat probe (Cortex Technology ApS, Aalborg, Denmark) was utilised to perform measurements of skin hydration. The assessments were conducted within a designated area (approximately 4 cm^2^) on the forearm eight times, and the mean was computed.

#### 2.4.4. Texture—Skin Roughness

Skin texture was assessed as the arithmetical mean roughness—Ra (µm) using topographic measurements [22] with an Antera 3D CS (Miravex Ltd., Dublin, Ireland) on a predetermined area on the left cheek.

#### 2.4.5. Wrinkles—Volume, Maximum Depth, Indentation Index

Antera 3D CS multispectral analyser (Miravex Ltd., Dublin, Ireland) topographic measurements were used to determine the severity of lateral periorbital wrinkles [22]. The volume of depressions (mm^3^), wrinkle maximum depth (mm) and wrinkle indentation index (a.u.) were evaluated. A medium filter, suitable for the assessment of fine to moderate wrinkles (0.5–2 mm in width), was used. Profilometry was conducted within a predetermined measurement zone on the lateral periorbital area on the side of the participant’s face with more pronounced wrinkles at baseline and with minimal presence of other irregularities, such as hyperpigmentation, that could impact the results.

### 2.5. Sample Size Calculations

The determination of the sample size was established based on findings from a prior investigation [23], where the effect size (η^2^) on the skin density resulting from dietary supplementation with a combination of HC (4 g) and coenzyme Q10 (50 mg) over 12 weeks was found to be 0.58 within groups, 0.13 between groups, and 0.33 within-between groups. In the present study, slightly higher doses of HC were used in combination with hyaluronic acid (HA) for a period extended by a further 4 weeks. Consequently, a slightly larger effect size was anticipated. To detect a within-between effect size of 0.33 with 80% power at a 5% statistical significance level, we determined a minimum of 25 participants per group.

### 2.6. Data and Statistical Analysis

To account for baseline variations between groups, analysis of covariance (ANCOVA) was used to compare the changes in dermal density and other parameters between the test groups accounting for baseline at midpoint and at the end of the intervention period. Dermal density and other observable parameters at the mid/endpoint of the intervention period were treated as dependent variables, whereas treatment vs. placebo group, time point, and their interaction were considered independent variables and baseline values as primary covariates. For all analyses, a two-tailed significance level of *p* < 0.05 was assumed. Means are presented with either a confidence interval (CI) or a standard deviation (SD).

After ANCOVA was used for significance testing and model marginal means were calculated, the results were transformed into absolute and relative intervention effects in comparison to the placebo, following Vickers’ recommended methodology [24]. The intervention effect (%) was calculated for each time point using the model-estimated mean and the following equation:

((post-treatment value_product) − (post-treatment value_placebo))/baseline value × 100%.

IBM SPSS Statistics for Windows, Version 27, was used for data management and statistical analyses (IBM Corp., Armonk, NY, USA). GraphPad Prism was utilised to generate the graphs (GraphPad Software, Version 9.5.1, La Jolla, CA, USA).

## 3. Results

**Figure 1:**
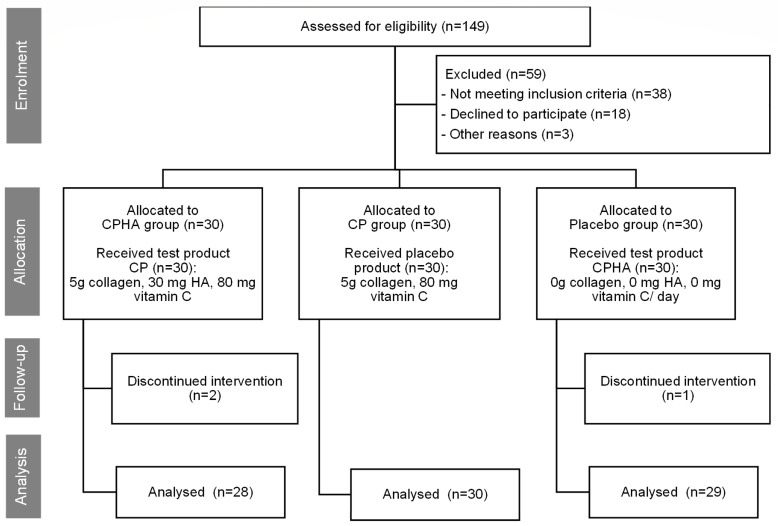
The trial design, subject’s allocation, and their progression throughout the trial.

Initially, a total of 149 women were evaluated for eligibility. Of these, 38 failed to meet the inclusion criteria, and 21 chose not to participate or withdrew for personal reasons prior to the start of the study. Of the 90 subjects enrolled, 87 completed the entire 16-week trial, distributed across the groups as follows: placebo group (29 subjects), CPHA group (28 subjects), and CP group (30 subjects). There were three drop-outs, one in the placebo group and two in the CPHA group, all for personal reasons. Throughout the study, no adverse events or side effects were documented. The Consolidated Standards of Reporting Trials (CONSORT) flow diagram [25] illustrates the trial design and the progression of individuals throughout the trial (Figure 1).

Table 1 presents the baseline features of the participants who successfully concluded the 16-week trial and were thus included in the analysis. Caucasian women (Fitzpatrick skin phototype I–IV) following an omnivorous diet were included in the study, with an average age of 52.2 ± 6.5 years, a mean body mass index (BMI) of 24.7 ± 3.9 kg/m^2^, and no significant differences between them at baseline.

Supplementary Table S1 includes the descriptive statistics for all variables regarding all test groups at baseline and follow-ups. Table 2 presents the results of the ANCOVA analysis. The change over time of interventions and comparisons to the placebo are reported using model marginal means with a 95% CI, while extended results of contrasts testing for differences of interventions compared to the placebo are included in Supplementary Table S2. Additionally, Table 2 presents results from statistical analysis describing the differences between the test products and the placebo accounting for baseline as covariate, while no significant differences were observed between CP and CPHA products within each time period.

**Figure 2:**
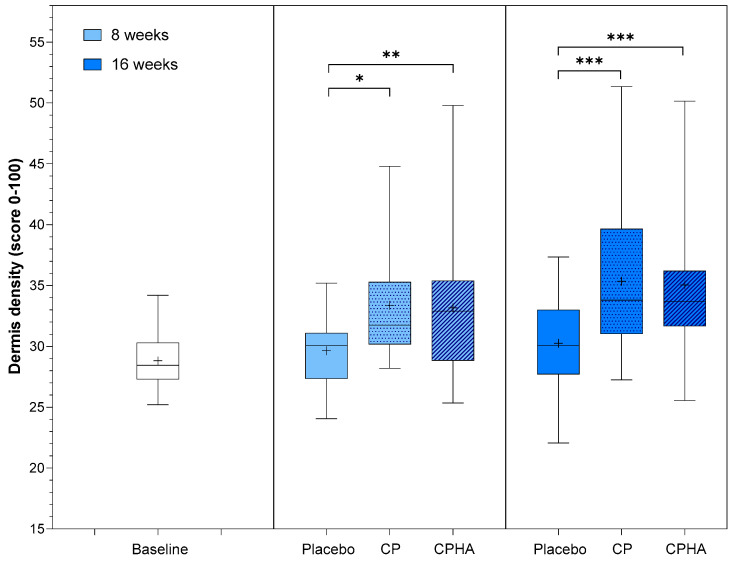
Dermis density before (baseline) and after 8- and 16-weeks of supplementation with study products. The boxplot shows the mean (+), median (−), and max-min whiskers. Absolute levels and significant differences (* *p* < 0.05, ** *p* < 0.01, *** *p* < 0.001) within time points between interventions are presented.

**Figure 3:**
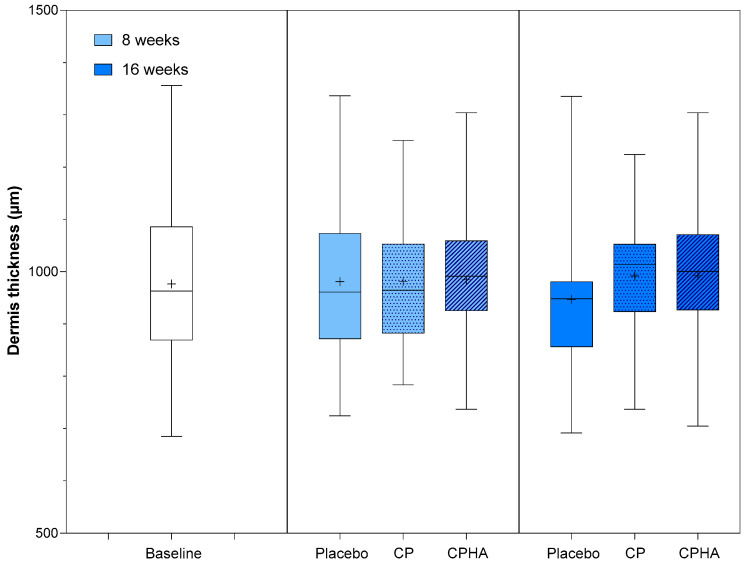
Dermis thickness before (baseline) and after 8 and 16 weeks of supplementation with study products. The boxplot shows the mean (+), median (−), and max-min whiskers. Absolute levels and differences within time points between interventions are presented.

**Figure 4:**
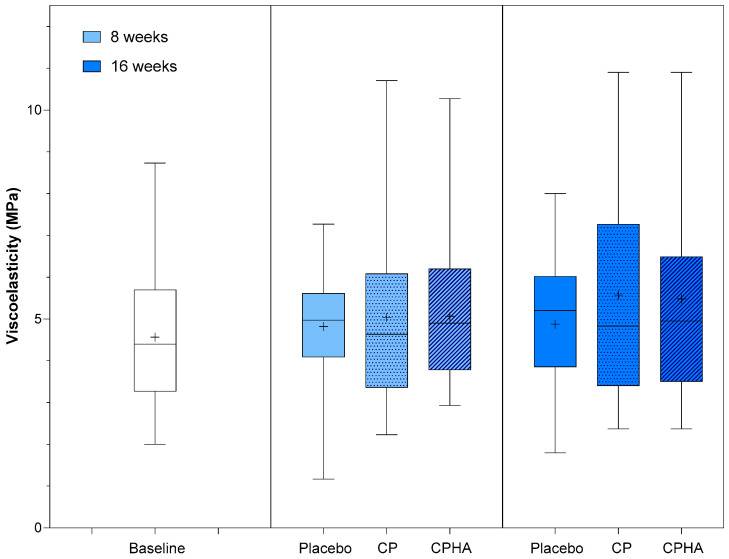
Viscoelasticity before (baseline) and after 8 and 16 weeks of supplementation with study products. The boxplot shows the mean (+), median (−), and max-min whiskers. Absolute levels and differences within time points between interventions are presented.

**Figure 5:**
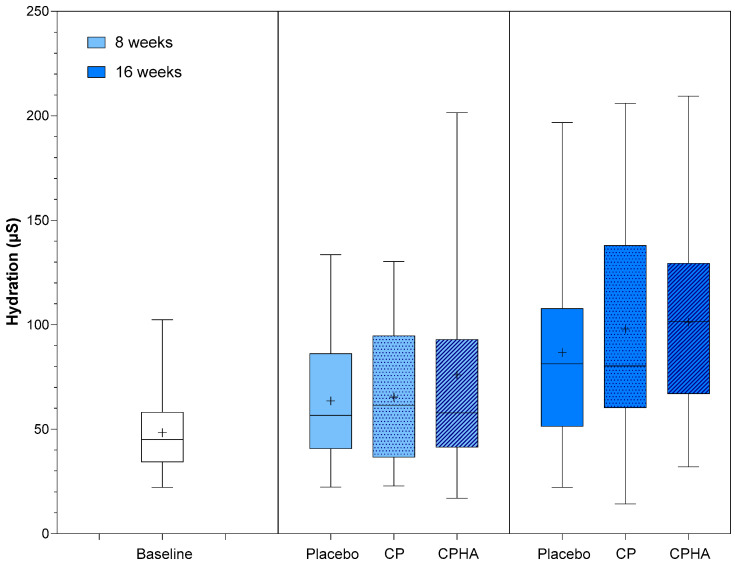
Skin hydration before (baseline) and after 8 and 16 weeks of supplementation with study products. The boxplot shows the mean (+), median (−), and max-min whiskers. Absolute levels and differences within time points between interventions are presented.

**Figure 6:**
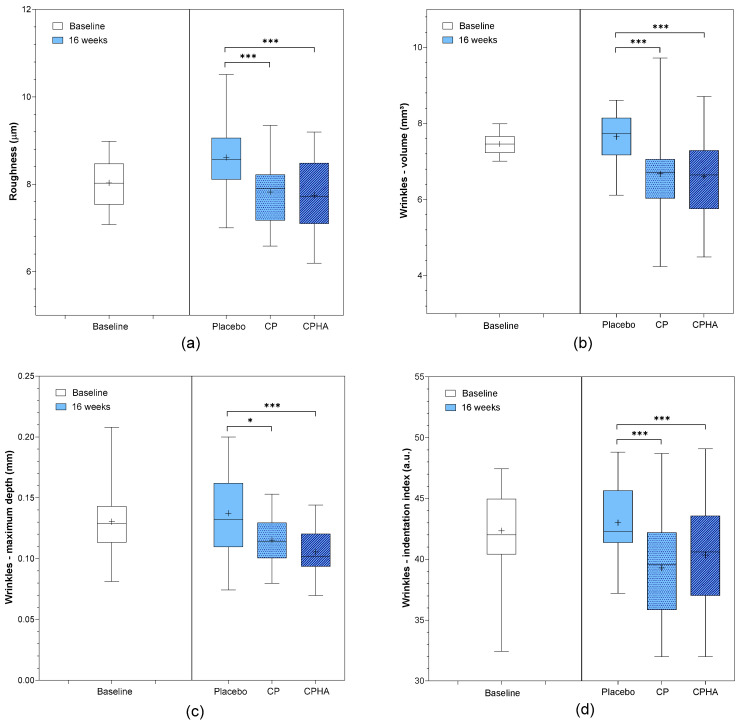
(**a**) Skin roughness, (**b**) wrinkles—volumes, (**c**) wrinkles- maximum depth and (**d**) wrinkles—indentation index before (baseline) and after 16 weeks of supplementation with study products. The boxplots show the mean (+), median (−), and max-min whiskers. Absolute levels and significant differences (* *p* < 0.05, *** *p* < 0.001) within time points between interventions are presented.

Figure 2, Figure 3, Figure 4, Figure 5 and Figure 6 represent the descriptive analysis of the variables and statistical comparisons between products after 8-(where applicable) and 16-weeks of supplementation. The baseline on the figure depicts data for subjects of all groups before intervention.

The significance levels, determined through the utilisation of ANCOVA, represent the pairwise comparisons among study groups. Extended results regarding absolute and relative effects in comparison to the placebo are included in Supplementary Table S3.

### 3.1. Dermis Density

At baseline, dermis density was comparable across all three groups (*p* = 0.73; see Table 1). The model-adjusted baseline score value was 28.82, and at 8- and 16-week follow-ups, density in the placebo group had not changed substantially, reaching 29.93 (95% CI 28.41–31.45) and 30.45 (95% CI 28.62–32.29), respectively (Table 2). Figure 2 shows the descriptive analysis of dermis density and statistical comparisons between products after 8- and 16 weeks of supplementation.

For both test products (CP and CPHA), a significant increase in density compared to the placebo was already observed at T8, and it was even more pronounced for both at the 16-week follow-up, as depicted in Table 2 and Figure 2.

The CP group showed an increase in skin density of 10.9% (to 33.07 (95% CI 31.57–34.56), *p* < 0.05) after 8 weeks, and throughout further intervention, the rise continued, with the intervention effect reaching 16.3% (to 35.16 (95% CI 33.35–36.97), *p* < 0.001) increase on average at 16-week follow-up, both significant in comparison to placebo. The observed results for the CPHA group were similar, with the intervention effect reaching an 11.3% significant increase on average (to 33.20 (95% CI 31.65–34.74); *p* < 0.01) in comparison to the placebo at 8-week follow-up, with further improvement reaching 16.0% (to 35.05 (95% CI 33.18–36.91), *p* < 0.001) after 16 weeks of intervention. No significant difference between the CP and CPHA groups was observed at either T8 or T16 (*p* = 0.86 and 0.79, respectively).

### 3.2. Dermis Thickness

Dermis thickness was comparable across all three groups at baseline (Table 1; Placebo: 972.3 ± 154.3 µm; CP: 978.0 ± 116.5 µm, CPHA: 979.8 ± 149.7 µm; *p* = 0.98). Figure 3 shows the descriptive analysis of dermis thickness and statistical comparisons between products after 8- and 16 weeks of supplementation, while model-adjusted values are represented in Table 2. The model-adjusted baseline value was 976.6 µm (Table 2), and in the placebo group at the 8 and 16-week follow-ups, the thickness was 984.0 µm (95% CI 955.8–1012.2) and 949.8 µm (95% CI 919.4–980.2), respectively.

Following 16 weeks of intervention, both test products showed an upward trend in skin thickness; the CP group exhibited a 4.1% increase (to 990.3 µm (95% CI 960.4–1020.2), *p* = 0.076) and the CPHA group a 4.2% increase (to 991.2 µm (95% CI 960.1–1022.2), *p* = 0.071)). However, for both groups, the rise from baseline failed to reach a statistically significant level in comparison to the placebo at either T8 and T16 and no significant differences between CP and CPHA groups were observed (*p* = 0.93 at T8 and 0.99 at T16).

### 3.3. Viscoelasticity

At baseline, viscoelasticity (VE) was comparable across all three groups (Table 1). Figure 4 represents the descriptive analysis of skin hydration and statistical comparisons between products after 8- and 16 weeks of supplementation, while model-adjusted values are represented in Table 2. The model-adjusted baseline value was 4.56 MPa (Table 2). In the placebo group, at T8, it was 4.78 MPa (95% CI 4.19–5.38), and at T16, it was 4.81 MPa (95% CI 3.96–5.65). During the intervention period, an increasing trend in VE was observed for both test products, but it did not reach superiority over placebo. At T16, the CP group showed an increase in viscoelasticity to 5.60 MPa (95% CI 4.77–6.43), reaching a 17.4% intervention effect, but it was not significant when compared to the placebo (*p* = 0.20). Similarly, the CPHA group’s VE increased to 5.46 MPa (95% CI 4.60–6.32), reaching a 14.3% intervention effect, but again, it was not significant when compared to the placebo (*p* = 0.14). At T8, the intervention effects were far less pronounced (6.6% and 1.9%, respectively, for CP and CPHA) and were also not significant for either of the test products. No significant differences between CP and CPHA groups were observed at either T8 or T16 (*p* = 0.97 and 0.94, respectively).

### 3.4. Skin Hydration

At baseline, skin hydration (µS) was comparable across all three groups (Table 1). Figure 5 represents the descriptive analysis of skin hydration and statistical comparisons between products after 8- and 16 weeks of supplementation, while model-adjusted values are represented in Table 2. The adjusted hydration baseline value was 48.3 µS, as can be observed from Table 2. During the intervention period, there was a noticeable upward trend in hydration levels for all products.

In the placebo group hydration reached 63.2 µS (95% CI 51.6–74.8) and 85.8 µS (95% CI 70.9–100.7) at the 8 and 16-week follow-up, respectively, while in the CP group it increased to 66.27 µS (95% CI 54.85–77.68; *p* = 0.64) at T8 and to 98.63 µS (95% CI 83.91–113.4; *p* = 0.19) at T16 and in the CPHA group it increased to 85.8 µS (95% CI 70.9–100.7; *p* = 0.22) at T8 and to 101.5 µS (95% CI 86.3–116.8, *p* = 0.18) at T16. However, changes for both test groups did not reach statistical significance when compared to placebo, and no significant differences between CP and CPHA products at either T8 or T16 were observed (*p* = 0.27 and 0.73, respectively).

### 3.5. Skin Roughness

At baseline, the arithmetical mean roughness (Ra) was comparable in all three groups (*p* = 0.76; Table 1). Figure 6a represents the descriptive analysis of Ra and statistical comparisons between products after 16 weeks of supplementation, while model-adjusted values are represented in Table 2. The Ra model adjusted baseline value was 8.05 µm. While in the placebo group, it reached 8.59 µm (95% CI 8.42–8.75) at the 16-week follow-up (Table 2), a perceivable and statistically significant change in roughness from baseline was observed in both test groups in comparison to the placebo, as presented also in Figure 6a. In the CP group, it dropped to 7.82 µm (95% CI 7.65–7.98), corresponding to a 9.6% improvement on average, and the effect was significant when compared to the placebo (*p* < 0.001). In the CPHA group, the result was similar, as the Ra reached 7.79 µm (95% CI 7.62–7.96) at 16-week follow-up, corresponding to an average 9.9% improvement, and the effect was also significant when compared to the placebo (*p* < 0.001). No significant difference between CP and CPHA groups was observed at the end of the supplementation period (*p* = 0.63).

### 3.6. Wrinkles

Wrinkle volume, maximum depth, and indentation index were the parameters used to assess wrinkle severity. All three parameters were comparable across the groups at baseline (Table 1). Figure 6b–d represents the descriptive analysis of wrinkle parameters and statistical comparisons between products after 16 weeks of supplementation, while model-adjusted values are represented in Table 2. At baseline, the model-adjusted wrinkle volume value was 7.46 mm^3^. The placebo group had a volume of 7.67 mm^3^ (95% CI 7.31–8.02) at the 16-week follow-up, while both test product groups showed a significant decrease (Table 2) when compared to the placebo (Figure 6b). The CP group experienced a 13.8% decrease in wrinkle volume vs. placebo (to 6.64 mm^3^ (95% CI 6.29–6.99), *p* < 0.001), and the CPHA group experienced a similar 13.9% decrease (to 6.63 mm^3^; 95% CI 6.27–7.00; *p* < 0.001), with no significant difference between CP and CPHA products (*p* = 0.72).

The model-adjusted wrinkle maximum depth was 0.130 mm at baseline. At the 16-week follow-up, the placebo group had a wrinkle max. depth of 0.135 mm (95% CI 0.127–0.142), while it was significantly reduced in both test product groups in comparison to the placebo group (Table 2, Figure 6c). The CP group experienced a 16.9% decrease in wrinkle maximum depth vs. the placebo (to 0.113 mm (95% CI 0.106–0.121), *p* < 0.05) on average, and the CPHA group experienced a significant 19.2% decrease (to 0.110 mm (95% CI 0.102–0.118), *p* < 0.001) vs. the placebo. There was no significant difference between the CP and CPHA groups (*p* = 0.13).

At baseline, the model-adjusted wrinkle indentation index was 42.3 a.u. At the 16-week follow-up, the indentation index for the placebo group was 43.3 a.u. (95% CI 42.4–44.3), while in both test groups, the indentation index significantly decreased compared to that of the placebo group (Table 2, Figure 6d). The CP group experienced an average 8.5% decrease in indentation index vs. the placebo (to 39.7 a.u. (95% CI 38.8–40.7), *p* < 0.05), and similarly, the CPHA group experienced an average 9.0% decrease (to 39.5 a.u. (95% CI 38.6–40.5), *p* < 0.001) vs. the placebo. There was no significant difference between the effectiveness of CP and CPHA products (*p* = 0.25).

## 4. Discussion

Skin ageing primarily arises from changes occurring in the dermis, the layer predominantly accountable for skin firmness and elasticity. The ability of substances to reach this deep layer is, therefore, crucial for yielding an enduring impact on the process of skin ageing. While topical skincare products often fail to reach the dermis adequately due to their limited skin penetration ability, oral supplementation provides an alternative route, facilitating the delivery of nutrients and other bioactive compounds via the bloodstream to the dermis, where they can exert effects.

Collagen constitutes the primary structural protein in the skin, comprising 75% of the overall weight of the ECM. The HC utilised in food supplements is derived through the hydrolysis of collagen found in natural sources, including beef, pork, fish, or chicken [26]. A recent review examined the mechanisms of collagen supplementation [27]. On digestion, collagen undergoes decomposition into amino acids, di- and tripeptides, which are then taken into the bloodstream via the gastrointestinal system and transported throughout the body, also reaching the skin. In the dermis, short peptides enhance the metabolic activity, migration and proliferation of fibroblasts and inhibit the activity of MMPs, resulting in increased synthesis and decreased loss of collagen, hyaluronic acid and other ECM components [13,27,28]. Furthermore, previous studies have demonstrated that collagen and its fragments have the capacity to inhibit the immune response against endogenous collagen. Mitigating inflammation and skin injury may contribute to enhanced skin health [27].

HA is an endogenous constituent found throughout the ECM, being particularly abundant in the skin, which comprises approx. 50% of the body’s total HA. It possesses moisturising characteristics and is essential for many physiological functions, such as tissue regeneration and repair, wound healing and inflammation responses [7,29,30]. In the skin, HA not only forms hydrogen bonds with water molecules but also controls the function of aquaporin-3, initiates endothelial cell proliferation and migration, and stimulates the production of collagen [31,32]. However, with increasing age, the concentration of HA in the skin diminishes considerably. These changes, along with declining collagen levels from the mid-30s, lead to a reduced dermal volume, elasticity, firmness, and hydration and manifest externally as sagging, wrinkles, and a rough texture to the skin [33,34].

Collagen peptides and hyaluronic acid supplementation are gaining attention. Various studies suggest the potential benefits of HC supplementation for skin health, but the effects vary depending on dosage and duration. In a study by Proksch et al. [35], consuming 2.5 g of HC daily for 8 weeks enhanced the production of the dermal ECM constituents, including elastin and procollagen type I, leading to a decrease in wrinkles’ severity around the eyes. Using similar interventions, Sugihara et al. [36] observed significant improvements in skin roughness, elasticity and hydration, while on the contrary, Proksch et al. [37] did not confirm any significant impact of even a double (5g) HC dose on skin roughness, hydration, or trans-epidermal water loss (TEWL, measure for skin barrier function). In a study conducted by Choi et al. [38] and Koizumi et al. [39], a 12-week supplementation with 3 g of HC enhanced skin elasticity and hydration. While Koizumi et al. [39] also noted a reduction in periorbital wrinkles, Choi et al. [38] failed to confirm enhancements in barrier function, and co-administration of 0.5 g of vitamin C alongside HC did not further amplify its effects on the skin. Asserin et al. [14] demonstrated that in women with dry skin, 8 weeks of supplementation with 10 g of fish or porcine HC improved hydration but had no effect on TEWL. After 12 weeks of supplementation, enhanced dermal collagen density was also detected, and ex-vivo experiments supported these findings by showing that collagen peptides stimulate collagen and glycosaminoglycan production. Miyanaga et al. [40] reported improved skin hydration and TEWL with daily 12-week consumption of either 1 g or 5 g of HC, but no enhancement of skin elasticity or thickness was observed. In our recent study [20], the effects of supplementation with different doses of HC (5 or 10 g daily) in combination with 1.5 g methylsulphonylmethane (MSM) or 10 g of HC over 12 weeks were investigated. While all the active products improved dermal density and skin roughness and diminished wrinkles, the products containing MSM improved skin roughness and thickness more effectively, and a higher concentration of HC in combination with MSM was essential for the enhancement of hydration. Conversely, the supplementation had no notable impacts on TEWL or elasticity with any of the products.

In several other studies, various active ingredients were combined with collagen. Pu et al. [19] recently conducted a meta-analysis exploring the impact of hydrolysed collagen supplementation on skin. It included 26 randomised, controlled trials (RCTs) involving over 1700 participants, mostly women, and showed positive effects of supplementation on elasticity and hydration. A previous meta-analysis by De Miranda et al. [17], which incorporated 19 RCTS and included approx. 1,100 participants altogether showed favourable outcomes of HC supplementation in reducing wrinkles. However, both meta-analyses were limited by the diversity of the data with regard to the collagen supplements employed, including origin, formulation, concentration as well as composition and variable HC daily dosages up to 12 g. Additionally, over half of the studies involved in those meta-analyses investigated supplementation with a combination of collagen and other bioactive constituents, potentially impacting the results.

While hydrolysed collagen peptides have shown beneficial effects on the skin in multiple studies, evidence from RCTs supporting the effects on the skin of supplementation with HA without other concomitant bioactives is very limited [7]. Oe et al. [41] investigated the influence of 12-week daily supplementation with 120 mg of HA of two different sizes (2 kDa and 300 kDa) on the skin of Japanese women. While both HA groups showed a trend to suppress wrinkles, the effect was significant (vs. the placebo) only for the 300 kDa group after 8 weeks; it was not significant at 12 weeks for either of the groups. Kawada et al. [42] investigated the influence of 6-week supplementation with 120 mg HA of two sizes (800 kDa or 300 kDA) on the skin of Japanese women with dry skin. During the supplementation period, there was no improvement vs. placebo in hydration or elasticity, but significant improvement in hydration was observed 2 weeks after the end of the intervention period in the 300 kDa group. In a study by Hsu et al. [43], a 12-week intake of 120 mg of HA in healthy Asians showed significant improvements in skin condition, including reduced wrinkles, increased hydration, decreased TEWL, and enhanced elasticity in the HA group compared to the placebo group. Michelotti et al. [44] tested a 4-week daily supplementation with 200 mg of HA with a large spectrum of molecular weights in Caucasian women. Results revealed notable improvements in skin hydration and TEWL, as well as a reduction in wrinkle depth and volume, accompanied by improved elasticity and firmness. These findings suggest that ingestion of HA may be beneficial for ageing skin. However, given that most of these studies mostly concentrated on the Asian population and many of them failed to demonstrate significant improvements when compared to a placebo, further research that includes Caucasians would be advantageous in investigating the impact of HA supplementation alone on the skin.

Based on the findings from our prior trial [20], which included either a daily intake of 10 g of HC with 80 mg of vitamin C or different doses of HC (5 g or 10 g) also combined with MSM, we wanted to further explore the efficacy of a lower dose of collagen. Hence, in the present double-blind, placebo-controlled study, we aimed to evaluate the efficacy of supplementation with a daily dosage of 5 g of HC with 80 mg of vitamin C alone (CP product) and in combination with 30 mg of HA (CPHA product) with a large spectrum of molecular weights over a longer, 16-week intervention period in women with ageing skin, focusing primarily on skin density. We also evaluated other skin parameters, including roughness, wrinkles, thickness, elasticity and hydration.

The study results demonstrate that both test products (CP and CPHA) effectively improved dermal density in women with skin ageing compared to the placebo. This was evident already after 8 weeks of intervention and continued to improve throughout the 16-week study period, reaching intervention effects of 16.3% and 16.0%, respectively. The achieved effects are comparable to those observed previously with a double HC dose over 12 weeks [20], but they were achieved over a period of 4 weeks longer. The observed enhancements in density also align with the findings of previous studies [14,23,45]. On the other hand, the results indicate that the addition of HA to collagen does not yield additional enhancements in skin density, as there was no notable difference in the efficacy between the two tested products.

After 16 weeks of supplementation, both test products (CP and CPHA) showed comparable effectiveness for reducing skin roughness (by 9.6% and 9.9%, respectively), as well as wrinkle severity, in comparison to the placebo. We observed significant reductions in wrinkle volume (by 13.8% and 13.9%, respectively), maximum depth (16.9% and 19.2%, respectively) and indentation index (8.5% and 9.0%, respectively) with no significant difference between them. The observed effects of the test products with regard to skin roughness as well as wrinkle severity are comparable to the results for a higher, 10 g HC dose after 12-week supplementation [20]. It can, therefore, be concluded that longer-term supplementation with a lower dose of collagen (5 g/day) is also sufficient for improvement in these parameters. On the other hand, as there was no significant difference in effectiveness between the CP and CPHA products, we could not confirm any beneficial effects of adding HA to collagen for improving skin texture or wrinkles.

Although an increasing trend of dermal thickness and viscoelasticity was observed for both test products, the changes in those parameters failed to reach a significance level for either product in comparison to the placebo. The observations for the CP product are consistent with our previous study [20], where 10 g of HC with 80 mg of vitamin C did not yield a significant effect on thickness or elasticity, and the addition of MSM to 5 or 10 g of HC was crucial for significant improvement in dermal thickness. On the other hand, in the present study, we did not observe any dermal thickness-enhancing effect from the addition of HA to collagen. Although the results of some previous studies using HA [43,44] indicated improvement in skin elasticity, we did not observe any additional improvement in elasticity with the addition of HA to collagen. Similarly, Kawada et al. [42] also failed to show the potential of hyaluronic acid (HA) for improving elasticity.

The results of skin hydration indicated rising tendencies for both test products, but the differences were not statistically significant when compared to the placebo. Observations for the CP product are consistent with our previous results, where supplementation with an even higher dose of HC did not reach significant improvement in hydration, and the addition of MSM was essential for the effect [20,23]. While some prior studies suggest that HA supplementation may enhance skin hydration [43,44], we were unable to confirm the beneficial effects of HA addition to collagen on skin hydration.

In our prior trial [20], it was suggested that an extended duration of HC supplementation could be advantageous for some parameters since 12 weeks might not be enough to discern notable nutritional impacts on the skin due to the typical epidermal skin cycle lasting 30–40 days in young, healthy people [5]. Hence, this study employed an extended 16-week intervention period. The extension yielded some advantages, particularly evident during the mid-term assessment, where a significant change in dermis density was observed after 8 weeks, while in our prior trial, it did not reach significance after 6 weeks of intervention despite using a double HC dose. A longer intervention period appears to confer benefits in terms of the magnitude of improvements in density and skin surface parameters (wrinkles, roughness) at the end of the study, as they were comparable to those observed previously utilising a double HC dose over a shorter intervention period. However, the extended intervention period did not significantly impact the results in terms of skin hydration and elasticity.

The potential of HC supplementation to penetrate the dermis and enhance collagen synthesis has been shown in several previous studies (Barati et al., 2020; Ohara et al., 2010; Yazaki et al., 2017) [13,27,46]. The present study further supports these observations, demonstrating that the effects of both tested products containing HC (CP and CPHA) extend beyond the epidermis; this highlights improvements in skin density, which manifest in reduced roughness and wrinkles. The observed advantageous effects indicate that the collagen in the tested products underwent efficient breakdown into biologically active peptides, which notably influenced dermal regeneration and visibly improved skin appearance. On the other hand, no discernible benefits regarding the assessed skin parameters were observed with the addition of HA to collagen. Therefore, we cannot confirm the ability of HA to function synergistically and further enhance the effects observed with HC supplementation.

This intervention study’s main strength was the thorough assessment of the impact of supplementation on a variety of skin parameters, as well as a comparison to a placebo. The observed placebo effects, particularly the rise in hydration levels, may be associated with seasonal climate changes. The study was conducted from the winter to the summer season, and it has been previously shown that increased relative humidity in summer compared to winter leads to an elevation in skin hydration, also resulting in a beneficial impact on skin elasticity and firmness [47,48]. Therefore, it was crucial to compare the test products with the placebo in order to mitigate the impact of seasonal factors.

We should also note some study limitations. All the test products included 80 mg of vitamin C, which may have influenced some of the investigated parameters [49], although prior studies [38] did not find such impacts even with a considerably higher dose of vitamin C (500 mg). The CPHA product contained a daily dose of 30 mg of HA, which is lower than the doses used in most of the prior studies using HA as a single active component, but is comparable to those studies that employed HA in combination with mixtures of other bioactives [50,51,52,53]. These studies also employed various molecular sizes of HA, which limits the direct comparability of the results.

## 5. Conclusions

In the present randomised, double-blind, placebo-controlled trial, the 16-week administration of liquid food supplements containing a daily dose of 5 g of collagen with 80 mg of vitamin C or their combination with 30 mg of hyaluronic acid was tested. The results showed beneficial effects of both interventions on the skin, improving dermis density as well as reducing skin roughness and wrinkle severity. However, there were no notable effects observed on skin elasticity or hydration following supplementation with either of the test products. As no superior effects of the addition of HA to collagen were observed on the assessed skin parameters, we cannot confirm the ability of HA to further improve the effects observed with collagen supplementation.

## Figures and Tables

**Table 1:** Participant characteristics at baseline and their comparison.

			Group				*p* Value
	Placebo (*n* = 29)		CP (*n* = 30)		CPHA (*n* = 28)		
	Mean	SD	Mean	SD	Mean	SD	
Age (years)	52.5	6.2	52.7	6.3	51.3	7.3	0.68
BMI (kg/m^2^)	24. 8	3.9	24.5	4.3	24.8	3.6	0.94
Density	28.5	2.4	29.1	2.5	28.8	3.5	0.73
Thickness (µm)	972.3	154.3	978.0	116.5	979.8	149.7	0.98
Viscoelasticity (Mpa)	4.5	1.6	4.5	1.3	4.2	1.2	0.96
Hydration (µS)	48.5	14.0	47.3	17.4	49.1	22.0	0.93
Roughness—Ra (μm)	8.07	0.48	7.98	0.53	8.05	0.68	0.76
Wrinkles							
- Volume (mm^3^)	7.44	0.29	7.51	0.24	7.43	0.25	0.46
- Maximum depth (mm)	0.133	0.032	0.133	0.017	0.123	0.016	0.18
- Indentation index (a.u.)	42.01	2.30	41.96	3.11	43.10	3.30	0.26

Notes: BMI: Body Mass Index; SD: Standard Deviation.

**Table 2:** Results of the model estimated marginal means (95% CI) of the intervention effect.

			Group		
Variables	Baseline Values	Follow-Up	Placebo Group	CP Group	CPHA Group
Density	28.82	8 weeks	29.93 (28.41–31.45)	33.07 (31.57–34.56) *	33.20 (31.65–34.74) **
		16 weeks	30.45 (28.62–32.29)	35.16 (33.35–36.97) ***	35.05 (33.18–36.91) ***
Thickness (µm)	976.6	8 weeks	984.0 (955.8–1012.2)	979.5 (951.7–1007.3)	983.4 (954.6–1012.2)
		16 weeks	949.8 (919.4–980.2)	990.3 (960.4–1020.2)	991.2 (960.1–1022.2)
Viscoelasticity (MPa)	4.56	8 weeks	4.78 (4.19–5.38)	5.08 (4.50–5.67)	4.87 (4.26–5.48)
		16 weeks	4.81 (3.96–5.65)	5.60 (4.77–6.43)	5.46 (4.60–6.32)
Hydration	48.31	8 weeks	63.16 (51.57–74.76)	66.27 (54.85–77.68)	75.38 (63.55–87.21)
		16 weeks	85.80 (70.85–100.74)	98.63 (83.91–113.4)	101.50 (86.25–116.75)
Roughness—Ra (μm)	8.05	16 weeks	8.59 (8.42–8.75)	7.82 (7.65–7.98) ***	7.79 (7.62–7.96) ***
Wrinkles					
- Volume (mm^3^)	7.46	16 weeks	7.67 (7.31–8.02)	6.64 (6.29–6.99) ***	6.63 (6.27–7.00) ***
- Maximum depth (mm)	0.130	16 weeks	0.135 (0.127–0.142)	0.113 (0.106–0.121) *	0.110 (0.102–0.118) ***
- Indentation index (a.u.)	42.34	16 weeks	43.33 (42.39–44.27)	39.73 (38.81–40.66) ***	39.53 (38.57–40.50) ***

Notes: * *p* < 0.05, ** *p* < 0.01, *** *p* < 0.001 indicate significant differences between the test products and the placebo accounting for baseline as covariate.

## Data Availability

The original contributions presented in the study are included in the article/Supplementary Materials. Further inquiries can be directed to the corresponding author/s.

## Supplementary Materials

The following supporting information can be downloaded at www.mdpi.com/xxx/s1.Supplementary Table S1. Outcome measures descriptive statistics for all study groups at baseline (T0) and follow-ups after 8 weeks (T8) (where applicable) and 16 weeks (T16) of intervention. Supplementary Table S2. Results of ANCOVA interaction effect between time and group (each intervention over placebo). Supplementary Table S3. Absolute and relative effects of intervention in comparison to the placebo at follow-ups.
