# Supplementary material for: The Effects of Dietary Supplementation with Collagen and Vitamin C and Their Combination with Hyaluronic Acid on Skin Density, Texture and Other Parameters: A Randomised, Double-Blind, Placebo-Controlled Trial
Source: Nutrients. 2024 Jun 17;16(12):1908. doi: 10.3390/nu16121908 (PMC11206740; doi:10.3390/nu16121908)
Supplement: Supplementary file 1 [file nutrients-16-01908-s001.zip › nutrients-3025822-supplementary.pdf]

Supplementary Materials

Supplementary Table S1. Outcome measures descriptive statistics for all study groups at baseline (T0) and follow-ups after 8 weeks (T8) (where applicable) and 16 weeks (T16) of intervention.

		Group					
		**Placebo**		**CP**		**CPHA**	
**Parameter**	**Time**	Mean	SD	Mean	SD	Mean	SD
Density (score 0-100)	T0	28.51	2.38	29.13	2.45	28.80	1.87
	T8	29.65	3.05	33.37	4.62	33.17	5.76
	T16	30.26	3.53	35.35	5.71	35.05	5.58
Thickness (μm)	T0	972.3	154.3	978.0	116.5	979.8	149.7
	T8	981.2	155.2	981.3	119.9	984.5	140.5
	T16	947.1	147.8	992.0	117.0	992.2	134.7
Viscoelasticity (MPa)	T0	4.63	1.27	4.53	1.62	4.54	1.79
	T8	4.82	1.33	5.04	2.00	5.06	1.70
	T16	4.87	1.72	5.40	2.49	5.48	2.31
Hydration (μS)	T0	48.52	13.99	47.34	17.35	49.14	21.96
	T8	63.56	31.33	65.35	33.38	75.95	48.29
	T16	86.76	45.57	97.94	51.16	101.25	44.94
Roughness - Ra (μm)	T0	8.07	0.48	8.08	0.53	7.98	0.62
	T16	8.61	0.75	7.83	0.68	7.75	0.78
Wrinkles - Volume (mm^3)	T0	7.44	0.29	7.51	0.24	7.43	0.25
	T16	7.65	0.64	6.68	1.14	6.61	1.01
Wrinkles - Maximum depth (mm)	T0	0.134	0.031	0.133	0.017	0.123	0.016
	T16	0.137	0.036	0.115	0.020	0.105	0.019
Wrinkles - Indentation index (a.u.)	T0	42.01	2.30	41.96	3.11	43.10	3.30
	T16	43.00	3.12	39.30	3.83	40.34	4.07

Notes: SD: standard deviation.

Supplementary Table S2. Results of ANCOVA interaction effect between time and group (each intervention over placebo).

Variable	Interaction effect (intervention x time)	CP		CPHA	
		F	p-value	F	p-value
Density	Level 2 vs. Level 1	12.91	**< 0.05**	8.44	**< 0.01**
	Level 3 vs. Level 1	14.8	**< 0.001**	14.14	**< 0.001**
Thickness	Level 2 vs. Level 1	0.049	0.826	0.001	0.977
	Level 3 vs. Level 1	3.279	0.076	3.402	0.071
Viscoelasticity	Level 2 vs. Level 1	0.478	0.492	0.079	0.779
	Level 3 vs. Level 1	1.665	0.202	2.218	0.142
Hydration	Level 2 vs. Level 1	0.218	0.643	1.517	0.224
	Level 3 vs. Level 1	1.72	0.195	1.846	0.18
Roughness - Ra	Level 2 vs. Level 1	39.83	**< 0.001**	43.62	**< 0.001**
Wrinkles					
- Volume	Level 2 vs. Level 1	17.56	**< 0.001**	21.257	**< 0.001**
- Maximum depth	Level 2 vs. Level 1	13.34	**< 0.05**	19.395	**< 0.001**
- Indentation index	Level 2 vs. Level 1	31.18	**< 0.001**	41.663	**< 0.001**

Notes: Level 1: baseline. level 2: 8-week follow-up. level 3: 16-week follow-up.

Supplementary Table S3. Absolute and relative effects of intervention in comparison to the placebo at follow-ups.

Variables	Time	Intervention effect		Intervention effect (%)	
		CP	CPHA	CP	CPHA
Density (score)	Follow-up 8 weeks	3.14*	3.27**	10.9*	11.3**
	Follow-up 16 weeks	4.71***	4.60***	16.3***	16.0***
Thickness (μm)	Follow-up 8 weeks	-4.5	-0.60	-0.5	-0.1
	Follow-up 16 weeks	40.5	41.40	4.1	4.2
Viscoelasticity (MPa)	Follow-up 8 weeks	0.3	0.09	6.6	2.0
	Follow-up 16 weeks	0.79	0.65	17.3	14.3
Hydration (μS)	Follow-up 8 weeks	3.11	12.22	6.4	25.3
	Follow-up 16 weeks	12.83	15.70	26.6	32.5
Roughness - Ra (μm)	Follow-up 16 weeks	-0.77***	-0.80***	-9.6***	-9.9***
Wrinkles					
- Volume (mm^3)	Follow-up 16 weeks	-1.03***	-1.04***	-13.8***	-13.9***
- Maximum depth (mm)	Follow-up 16 weeks	-0.022*	-0.03***	-16.9*	-19.2***
- Indentation index (a.u.)	Follow-up 16 weeks	-3.6***	-3.80***	-8.5***	-9.0***

Notes: *p<0.05. **p<0.01. ***p<0.001 for comparison of change from baseline at follow-ups between intervention and placebo.
